# Diversity of site-specific microbes of occlusal and proximal lesions in severe- early childhood caries (S-ECC)

**DOI:** 10.1080/20002297.2022.2037832

**Published:** 2022-02-13

**Authors:** Kausar Sadia Fakhruddin, Lakshman Perera Samaranayake, Rifat Akram Hamoudi, Hien Chi Ngo, Hiroshi Egusa

**Affiliations:** a Department of Preventive and Restorative Dentistry, University of Sharjah, Sharjah, UAE; b Division of Molecular and Regenerative Prosthodontics, Tohoku University Graduate School of Dentistry, Sendai-city, Japan; c The University of Hong Kong, Hong Kong Special Administrative Region, China; d Sharjah Institute for Medical Research, College of Medicine, University of Sharjah, Sharjah, UAE; e Division of Surgery and Interventional Science, University College London, London, UK; f Uwa Dental School, The University of Western Australia, Perth, Australia

**Keywords:** Severe-early childhood caries (S-ECC), dentine caries, occlusal-caries lesion, proximal-caries lesion, microbial diversity

## Abstract

**Background:**

Severe-early childhood caries (S-ECC) a global problem of significant concern, commonly manifest on the occlusal, and proximal surfaces of affected teeth. Despite the major ecological differences between these two niches the compositional differences, if any, in the microbiota of such lesions is unknown.

**Methods:**

Deep-dentine caries samples from asymptomatic primary molars of children with S-ECC (n 19) belonging to caries-code 5/6, (ICDAS classification) were evaluated. Employing two primer pools, we amplified and compared the bacterial *16S rRNA* gene sequences of the seven hypervariable regions (V2—V4 and V6—V9) using NGS-based assay.

**Results:**

Bray-Curtisevaluation indicated that occlusal lesions (OL) had a more homogeneous community than the proximal lesions (PL) with significant compositional differences at the species level (*p* = 0.01; R- 0.513). Together, the occlusal and proximal niches harbored 263 species, of which 202 (76.8%) species were common to both , while 49 (18.6%) and 12 (4.6%) disparate species were exclusively isolated from the proximal and occlusal niches, respectively. The most commonl genera at both niches included *Streptococcus, Prevotella*, and *Lactobacillus. S. mutans* was predominant in PL (*p* ≤ 0.05), and *Atopobium parvulum* (*p* = 0.01) was predominant in OL.

**Conclusions:**

Distinct differences exist between the caries microbiota of occlusal and proximal caries in S-ECC.

## Introduction

The single most common chronic disease condition of childhood is considered to be dental caries which affects 60–90% of all school children [1,2]. Severe early childhood caries (S-ECC) is a rapidly developing variant of dental caries. It is classified by the presence of a decayed, missing (due to caries), or filled tooth (dmft) index score of ≥ 4 (age 3), ≥ 5 (age 4), or ≥ 6 (age 5) [3]. S-ECC is a destructive disease and can lead to acute pain and sepsis, potential tooth loss, and poor quality of life of the affected individual. This is further exacerbated by poor nutrition and retarded school participation due to dental pain and infection [4]. Furthermore, S-ECC is a risk factor for caries of permanent teeth [5,6], and affected children are more likely to develop recurrent caries [4,7].

S-ECC, a disease of complex aetiology, is an outcome of a dysbiotic oral microbiome of polymicrobial plaque biofilm communities [8]. Such biofilm sustenance is assisted by recurrent sugar pulses derived from dietary carbohydrates frequently consumed by children [9,10]. Carboxylic acids produced by biofilm metabolic processes are the root cause of the dissolution of the hydroxyapatite enamel matrix [11,12]. If unabated, this destructive process, leads to the lesion progression into coronal dentin by a diverse group of microbiota co-existing in a dynamic ecological equilibrium within the biofilm [11,13].

It is known that the caries progression from enamel to dentine is associated with the compositional transformation of the microbiome with an assortment of proteolytic, amino-acid degrading microbes predominating in the deeper dentinal lesions, in addition to the saccharolytic acidogenic/aciduric microbes [13]. Such differences in the enamel and dentinal caries progression may be due to their histological and structural differences. Compared to enamel, dentin is less mineralized, with mineral densities for normal enamel and dentin ranging between 2,170–3,100 mg/ml and 1,290–1,530 mg/ml, respectively [14,15]. About a third of the dentine matrix comprises easily soluble organic material – primarily collagen, constituting approximately 90% of the organic matrix [16].

Site-specific microbial colonization, referred to above is also dictated by contributory factors, including the tooth topography, nutrient availability, pH and redox (Eh) conditions, and salivary flow rates [17]. The ecosystem of such cavitated deep dentinal carious lesions is unique in that the sheltered niches are almost unreachable in routine oral hygiene measures.

In terms of the predominant microbiota of dental caries, both streptococcal and lactobacillus genera have long been recognized to play a critical role, although recent work indicates clearly that fungi, predominantly *Candida* species, are prevalent in deep cavitated lesions [18,19]. Indeed, in a recent study, we have unequivocally demonstrated the profusion of candidal species in S-ECC [20]. Furthermore, the current consensus is that caries, in general, is not caused by specific organisms, such as *Streptococcus mutans* and lactobacilli, but by a polymicrobial consortium of cariogenic species. For instance, recent studies have identified other genera such as *Bifidobacterium, Scardovia, Veillonella*, and *Actinomyces* species as significant contributors to ECC [13,21–25].

S-ECC is common on both the occlusal and proximal surfaces of teeth. While the retentive occlusal pits and fissures of enamel are the common sites of early lesion formation, interproximal surfaces too are particularly susceptible to S-ECC [26]. This may be due to the proximal habitats being protected physically and under-exposed to the regular flushing action of saliva, masticatory forces, and tongue movements compared to the occlusal habitats [26,27]. Last but not least, the detection of proximal caries lesions is challenging even for experienced professionals unless examined with extreme care, using imaging techniques [28].

In patients with a high-caries risk, the proximal dentinal lesions progress even after receiving micro-invasive treatment [29]. Due to these intrinsic differences between occlusal and proximal caries, it is likely that they harbor a unique plaque microbiota. Site-specific microbial colonization has been demonstrated in an elegant study by Simon-Soro et al. (2013), even in healthy tooth sites. They reported considerable differences in microbial composition between teeth at different intra-oral locations and between different surfaces of the same tooth. For instance, they noted 40% to 70% *Streptococcus* species on the vestibular surfaces, in contrast to their minimal prevalence on the lingual surfaces of incisors and canines [30].

We, therefore, hypothesized that the core microbiota of occlusal and proximal caries in S-ECC is likely to be fundamentally different. Therefore, this study aimed to compare the microbial profile of occlusal and proximal caries in S-ECC using a next-generation sequencing (NGS) assay. The current report, to our knowledge, is the first to describe the differences in the microbiota of occlusal and proximal caries in S-ECC.

## Material and methods

### Ethics statement

The Research Ethics Committee, University of Sharjah, approved the protocol (REC-18-02-18-03) of the study. Nineteen children, aged-48-months to 72-months, attending routine pediatric teaching clinics at the University Dental Hospital Sharjah (UDHS), United Arab Emirates, were invited to participate in the study. Before the oral examination, verbal and written informed consent were obtained from each child participant recruited in the study.

### Study subjects and dental examination

A complete dental examination was carried out for all healthy, cooperative participants. Children with five or more decayed teeth and at least two asymptomatic primary molars in different mouth quadrants with either occlusal or proximal caries lesions involved were selected.

### Caries diagnosis

The World Health Organization (WHO) criterion of decayed, missing, and filled (dmft) tooth index was used to record the caries status and to ascertain the severity of cavitated lesions as either caries code-5 or code-6, according to ICDAS- caries criteria [31]. One trained pediatric dentist (KSF) conducted a clinical examination and sample collection throughout the study.

The examiner determined the severity of cavitated lesions according to ICDAS classification, viz. code −5 being a distinct cavity with visible dentin involving less than half of the tooth surface, and code −6, a distinct and extensive cavity with visible dentin affecting more than half of the surface.

The exclusion criteria were children on antibiotics over the last 4-weeks before sample collection, those wearing orthodontic appliance/s or with congenital tooth anomalies, or any likelihood of pulp exposure during the caries excavation process. Further, dentin samples from endodontically treated teeth or when gingival bleeding contaminated the cavity during the sample collection process were also excluded.

### Sample collection and DNA extraction

A total of 38 infected-dentin samples from 19 children were aseptically collected by a single trained collector (KSF) from an occlusal and a proximal, symptom-free, caries active, deep-dentin lesions belonging to ICDAS caries-code 5 and code 6.

Dentin samples were collected using a sterile spoon excavator after cleaning and drying the cavities with a prophy brush without using prophy paste, as described in a previous study [8]. The collected samples were placed in an Eppendorf centrifuge tube (1.5 ml) containing 300 µl of phosphate-buffered saline (PBS) and immediately frozen at −20°C until further use for NGS run.

DNA extraction of the collected infected-dentin samples was performed using MasterPure™ Complete DNA and RNA Purification (Epicenter, USA), following the manufacturer’s instructions. The extracted DNA’s quality and quantity were assessed using a Colibri Microvolume Spectrometer (Titertek-Berthold Detection Systems GmbH, Germany). Extracted DNA samples were considered pure if the A260/280 ratio was higher than 1.8, and the A260/230 estimates were in the range of 1–2.2. Intact dsDNAs were measured using Qubit-DNA quantitation (Qubit4 Fluorometric Quantitation, Thermo Fisher Scientific, USA) before NGS.

### 16S rRNA gene amplicon sequencing of the caries-dentin samples

Bacterial samples from deep-infected carious dentin were sequenced using the Ion S5XL semi-conductor sequencing system (Thermo Fisher Scientific, USA). For the preparation of amplicons, a combination of the two sets of primers [primer set-1 V2-4-8 and primer set-2 V3-6, 7–9] (Ion 16S metagenomics kit) were used for selectively amplifying the corresponding hypervariable regions of the 16S rRNA region of bacteria [32]. For each sample, two reactions were prepared (one with each set of primers), using ‘water’ as negative control and ‘diluted *Escherichia coli* DNA’ as negative-positive control. The amplified products were then purified using Agencourt AMPure XP Reagent, Beckman Coulter. Thus, purified amplicons were quantified using DNA highly sensitive reagents in the Qubit 3 fluorometer, and ~10 ng of the purified amplicon from each primer set were pooled. The library was prepared utilizing the Ion Plus Fragment Library Kit (Catalog #4,471,252, Thermo-Fisher Scientific) as per the manufacturers’ instruction.

In brief, the pooled amplicons were end-repaired using end repair enzyme followed by purification using 1.8 volumes of Agencourt AMPure XP Reagent. The purified end-repaired amplicons were ligated with adapters and unique barcodes followed by nick repair. Thus, the prepared library was purified using 1.4 volumes of Agencourt AMPure reagent, and the purified library was quantified using the Ion universal library quantitation kit (Thermo Fisher Scientific). The libraries were further diluted to 10 pM and pooled equally with 16 individual samples per pool and were amplified using emulsion PCR on Ion One Touch2 instruments (OT2) followed by enrichment on Ion One Touch ES according to the manufacturer^,^s instruction. Thus, prepared template libraries were then sequenced on the Ion S5 XL Semiconductor sequencer using the Ion 520 Chip.

### 16S rRNA data analysis and taxonomy assignment

The metagenomics data were analyzed using the Ion Torrent Software Suite version 5.4. Following sequencing, the individual sequence reads were filtered to remove low-quality sequences. Quality control of sequencing reads retained sequences with a length between 120 and 350 bp. All quality-approved, trimmed, and filtered data were exported as unaligned BAM files to the Ion Reporter software (version 5.10) (Thermo Fisher Scientific) [33] where the sequences from the polymicrobial dentin samples where aligned to the following databases; Greengenes (version 13.5), MicroSEQ and the 16S reference library (version 2013.1) to generate the BAM files.

To calculate downstream diversity measures using alpha and beta diversity metrics, 16S rRNA operational taxonomic units (OTUs) were defined at ≥ 97% sequence homology. After excluding singleton reads, using UPARSE [34], OTUs were constructed by clustering reads with a minimum pair-wise identity of 97%. All clustered-quality-checked reads were then mapped to each OTU with 97% similarity using UPARSE. Using the ChimeraSlayer utility, chimeras were identified and removed from the analysis [35]. All reads were classified to the lowermost possible taxonomic rank using QIIME and a reference dataset from the HOMD [36]. Through the vegan package of R (R-Vegan 2.4–2 package), the number of OTUs was calculated following rarefaction to 3,000 reads/sample. It was employed as an index of bacterial diversity in the present study. Following this, we proceed with alpha diversity (Shannon) and beta diversity (Bray-Curtis) metrics after normalizing abundance of < 1% counts.

### Statistical analyses

To evaluate the significant differences between occlusal and proximal dentin samples, continuous variables were compared using the *t-*test. Microsoft Excel 2019 (Microsoft Office, 2019) was used for statistical calculations. A Bray-Curtis dissimilarity matrix table was used as input for Multi-Dimensional Scaling (MDS). To test whether occlusal and proximal groups samples significantly differed in their microbiota, we used the ANOSIM (Analysis of Similarities) test in R. A *p-*value of ≤ 0.05 was considered significant.

## Results

Deep dentinal caries samples derived from 19-occlusal and 19-proximal sites from asymptomatic primary molars of 19 children (mean-age 5.1 ± 0.76 years) with S-ECC were evaluated.

Before quality filtering, the *16S rRNA* gene sequencing platform yielded a total of 5,088,337-proximal and 4,366,100-occlusal reads from the sequencing run containing V2-V9 (except V5) regions of the bacterial *OTU* gene. Two hundred and eighty-three OTUs were obtained from the V2-V4 region and 397 from the V5-V6 region. After the quality filtering of all sequences (i.e. the removal of chimeras and de-noising -up to OTU assignments with > 97% identity), the Ion Torrent sequencing platform generated an average of 288,144 reads/proximal and 229,182 reads/occlusal samples, with the mean length of 219 and 177 bp, respectively.

**Figure 1.**
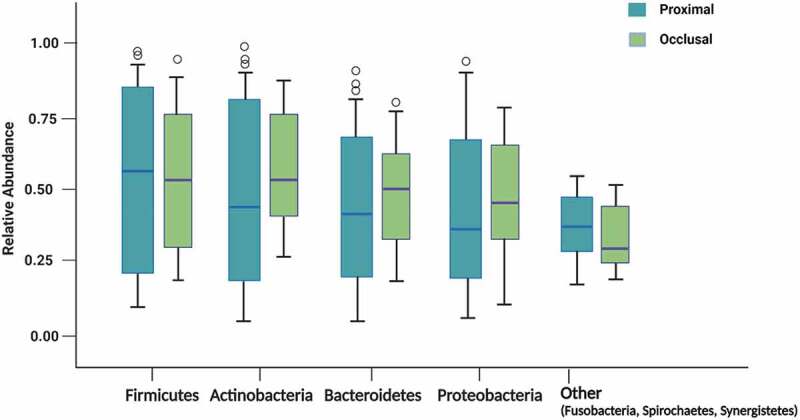
Box and whiskers plot representing the relative abundance of dominant phyla (*Firmicutes, Actinobacteria, Bacteroidetes, Proteobacteria*) and minor occurrence phyla including *Fusobacteria, Spirochaetes*, and *Synergistetes* by occlusal (green) and proximal (blue) caries samples. The upper and lower end of the box representing the first (Q1) and third (Q3) quartiles. The horizontal lines in the box represent the median. The whiskers representing 1.5 x interquartile range, and the circles represent outliers.

At the phylum level, both the occlusal and proximal caries microbiota were dominated by *Firmicutes* followed by *Actinobacteria, Bacteroidetes*, and *Proteobacteria* in descending order of predominance. In addition, a low proportion of *Fusobacteria, Spirochaetes*, and *Synergistetes* phyla also habituated the occlusal and proximal cavities (Figure 1).

On the other hand, the predominant genera in both the occlusal and proximal niches were *Streptococcus, Prevotella*, and *Lactobacillus*, with 33, 27, and 22 species each, respectively, (Figure 2a-e).**Figure 2.**
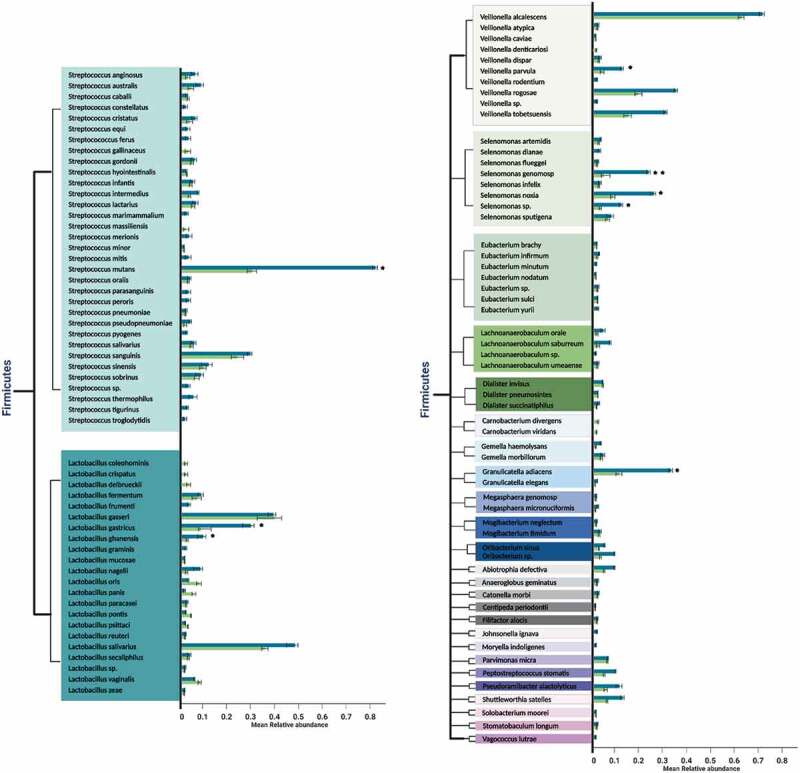
a Mean relative abundances of differentially prevalent genera belonging to most dominant phylum *Firmicutes* in the occlusal and proximal caries samples. Error bars represent standard deviation, indicative of the variation between occlusal samples or proximal samples in relation to the frequency of occurrence of the species. Taxon showing a significant difference in abundance between proximal and occlusal samples is marked by (*p* ≤ 0.05* and *p* = 0.01**). A *p-*value was obtained using *t-* test. A different color indicates each genus in the left column; occlusal caries samples are green, and proximal caries samples are blue on the right column. (2b). Mean relative abundances of differentially prevalent genera belonging to the phylum *Actinobacteria* in the occlusal and proximal caries samples. Error bars represent standard deviation, indicative of the variation between occlusal samples or proximal samples in relation to the frequency of occurrence of the species. Taxon showing a significant difference in abundance between proximal and occlusal samples is marked by asterisks (*p* ≤ 0.05* and *p* = 0.01**). A *p-*value was obtained using *t-*test. A different color indicates each genus in the left column; occlusal caries samples are in green, and proximal caries samples are in blue on the right column. (2c) Mean relative abundances of differentially prevalent genera belonging to phylum in the occlusal and proximal caries samples. Error bars represent standard deviation, indicative of the variation between occlusal samples or proximal samples in relation to the frequency of occurrence of the species. Taxon showing a significant difference in abundance between proximal and occlusal samples is marked by asterisks (*p* ≤ 0.05* and *p* = 0.01**). A *p-*value was obtained using *t-*test. A different color indicates each genus in the left column; occlusal caries samples are in green, and proximal caries samples are in blue on the right column. (2d) Mean relative abundances of differentially prevalent genera belonging to phylum *Proteobacteria* in the occlusal and proximal caries samples. Error bars represent standard deviation, indicative of the variation between occlusal samples or proximal samples in relation to the frequency of occurrence of the species. Taxon showing a significant difference in abundance between proximal and occlusal samples is marked by asterisks (*p* ≤ 0.05* and *p* = 0.01**). A *p-*value was obtained using *t-*test. Different color indicates each genus in the left column; occlusal caries samples are in green, and proximal caries samples are in blue on the right column. **2e**. Mean relative abundances of differentially prevalent genera belonging to minor phyla *Fusobacteria, Spirochaetes*, and *Synergistetes* in the occlusal and proximal caries samples. Error bars represent standard deviation, indicative of the variation between occlusal samples or proximal samples in relation to the frequency of occurrence of the species. Taxon showing a significant difference in abundance between proximal and occlusal samples is marked by asterisks (*p* ≤ 0.05* and *p* = 0.01**). A *p-*value was obtained using *t-*test. Different color indicates each genus in the left column; occlusal caries samples are in green, and proximal caries samples are in blue on the right column.

**Figure 2.**
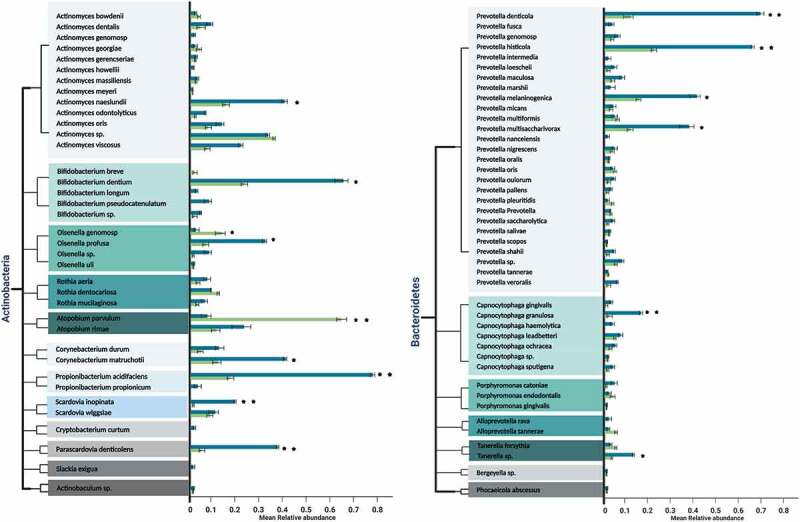
Continued.

**Figure 2.**
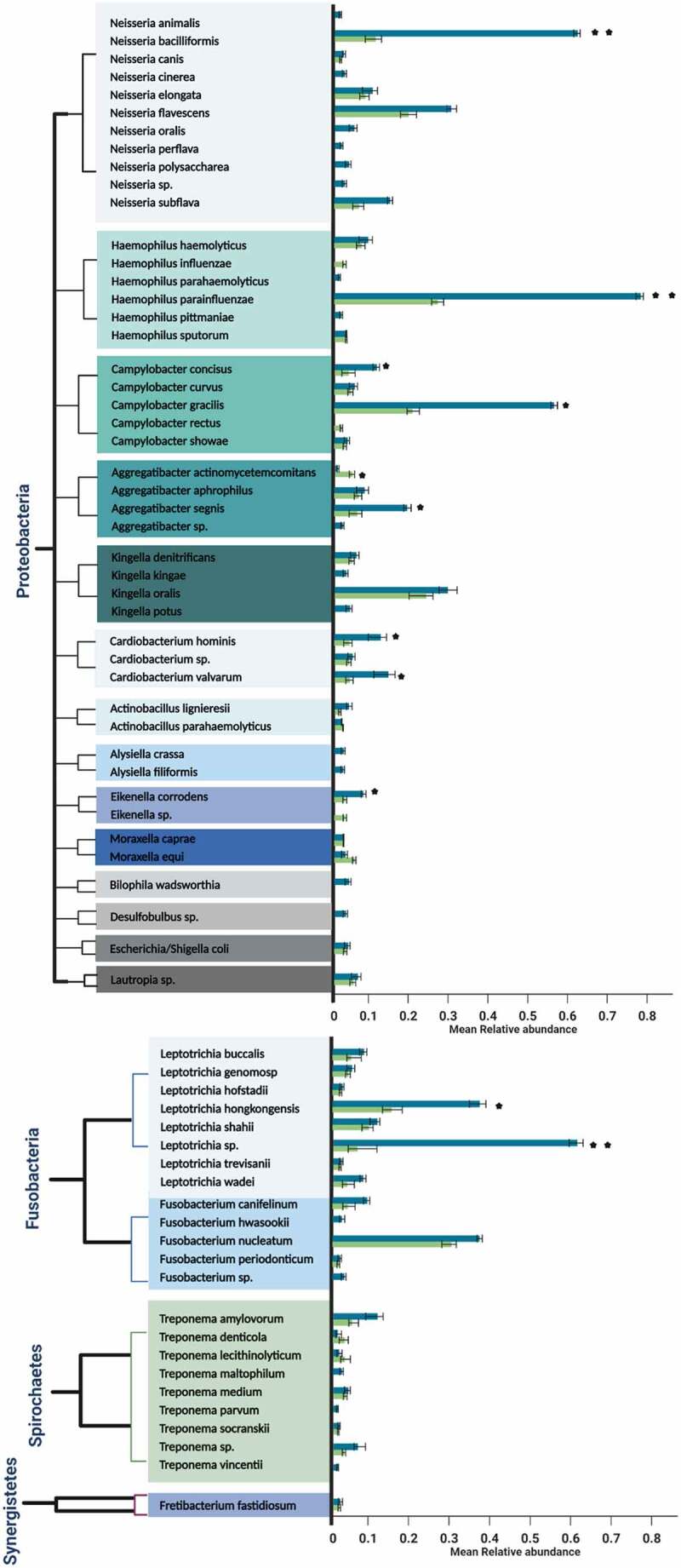
Continued.

**Figure 3.**
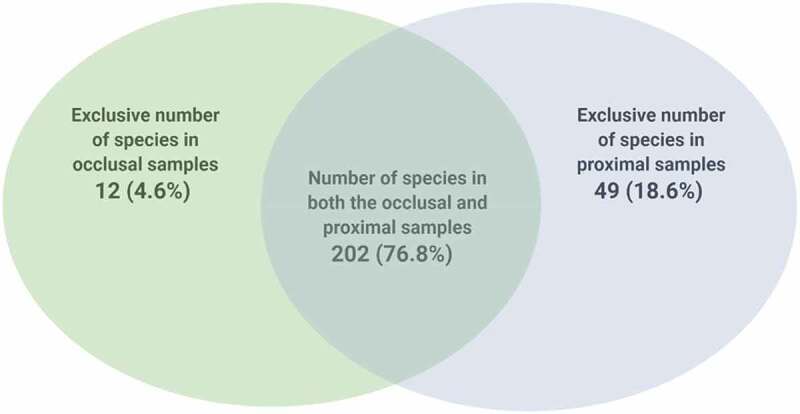
Species occurrence in 19 occlusal and 19 proximal caries samples in a cohort of 19 children with S-ECC.

A total of 253 predominantly acidogenic and acidophilic species inhabited both the occlusal and proximal eco-niches, some of which were found solely in either the occlusal or proximal cavities. For instance, both the occlusal and proximal niches harbored 263 species, of which 202 (76.8%) species were common to both locales, while 49 (18.6%) and 12 (4.6%) disparate species were exclusively isolated from the proximal and occlusal niches, respectively (Figure 3).

*S. mutans* was the most prevalent species isolated, but with a significant presence in the proximal cavities (*p* ≤ 0.05). In addition, both the proximal and occlusal locales had an abundance of *Veillonella alcalescens* (*p* ≥ 0.05).

Significant differences in both the bacterial composition and diversity between the occlusal and proximal caries lesions were noted (*p* < 0.05). Proximal caries lesions exhibited a greater diversity in the overall microbial population compared to the occlusal locales. We observed 31 highly prevalent species in proximal caries lesions, in contrast to only three species in the occlusal lesions (*p* < 0.05).

*Atopobium parvulum*, belonging to phylum *Actinobacteria*, was the most significantly abundant resident of the occlusal in comparison to proximal cavities (*p* = 0.01). In terms of the minor abundance species, *Olsenella* genomosp. and *Aggregatibacter actinomycetemcomitans*, belonging to phyla *Actinobacteria* and *Proteobacteria*, were significantly higher (*p* ≤ 0.05) in the occlusal, in comparison to the proximal niche.

Proximal niches harbored several significantly prevalent caries-associated microbiota, in descending quantitative frequency: *Haemophilus influenzae* (*p* = 0.01) followed by *Propionibacterium acidificiens* (*p* = 0.01), *Prevotella denticola* (*p* = 0.01), *Prevotella histicola* (*p* = 0.01), *Bifidobacterium dentium* (*p* ≤ 0.05), *Prevotella melaninogenica* (*p* ≤ 0.05), *Leptotrichia* sp. (*p* = 0.01), *Corynebacterium matrochotii* (*p* ≤ 0.05), *Lactobacillus gastricus* (*p* ≤ 0.05), and lastly, *Prevotella multisaccahrivorax* (*p* ≤ 0.05). Further detailed microbial analysis showed the moderate presence of *Neisseria bacilliformis* (*p* = 0.01) and *Campylobacter gracilis* (*p* ≤ 0.05) in the proximal cavities compared to occlusal niches.

**Figure 4.**
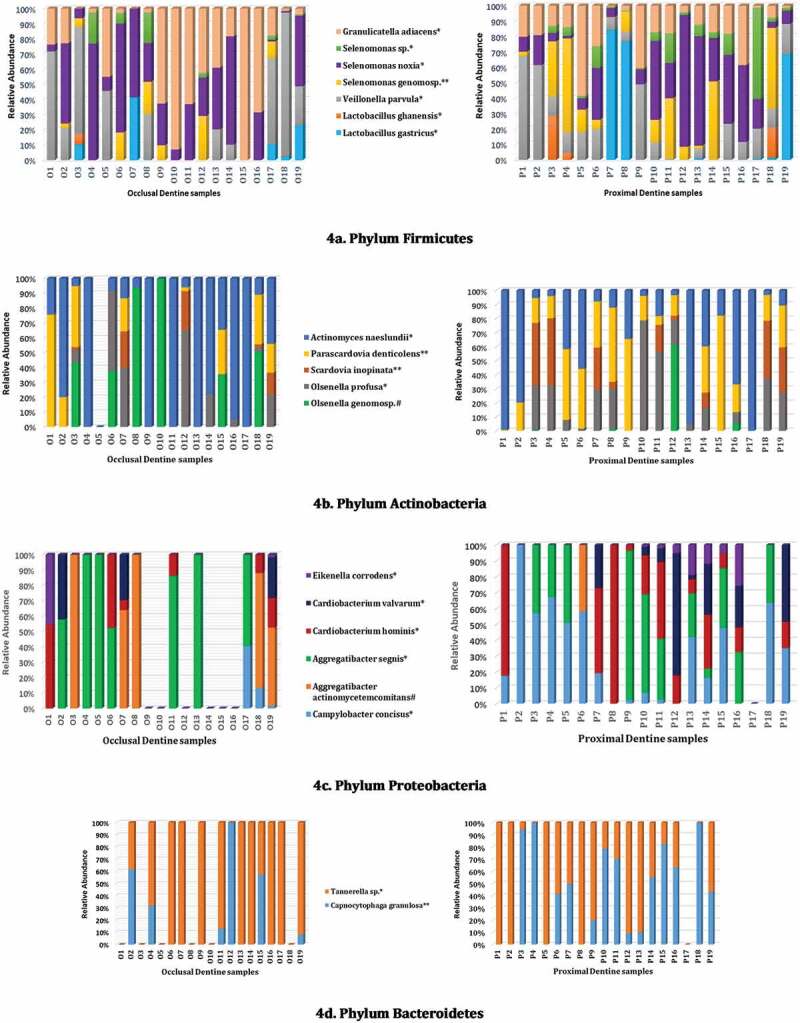
(a-d) Relative abundance of low occurrence taxa across both occlusal and proximal caries samples of phyla *Firmicutes* (a) *Actinobacteria* (b) *Proteobacteria* (c) and *Bacteroidetes* (d) Percentages for each taxon represent the median abundance values for each sample. Taxon showing a significant difference in abundance between proximal or occlusal dentine samples are marked *p* ≤ 0.05* and *p* = 0.01** and *p* ≤ 0.05#, respectively. *p-*value was obtained using *t-*test.

In terms of abundance, six different species (*Lactobacillus ghanensis, Veillonella parvulum, Selenomonas* genomosp., *Selenomonas noxia, Selenomonas* spp., and *Granulicatella adiacens*) from the most dominant phylum *Firmicutes* were noted in proximal caries (*p* ≤ 0.05; Figure 4a). In addition, the latter niches harbored significantly more species of *Proteobacteria*l (*Campylobacter concisus, Aggregatibacter segnis, Cardiobacterium hominis, Cardiobacterium valvarum*, and *Eikenella corrodens*) and *Actinobacteria*l (*Olsenella profusa, Scardovia inopinata, Parascardovia denticolens* and *Actinomyces naeslundii*) species (*p* ≤ 0.05; Figure 4b-c). These were in addition to *Capnocytophaga granulosa* (*p* = 0.01) and *Tanerella* sp. belonging to phylum *Bacteroidetes*, which were significantly more common in the proximal cavities (*p* ≤ 0.05; Figure 4d).

**Figure 5.**
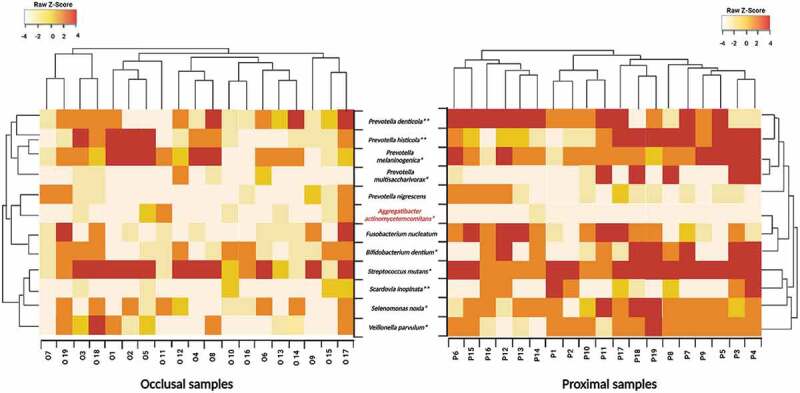
Heatmap showing 12 species belonging to eight genera with known collagenolytic attributes present in the proximal and occlusal caries samples of children with S-ECC. The heat map was generated using the g-plots package by clustering proximal and occlusal infected dentin samples based on the distribution and relative abundance of the microbial species with proteolytic potential. Clustering shows the similarity of samples. The heat map scale displays the row Z score (Z score = [actual relative abundances of a species in each sample − mean relative abundance of the same species in the proximal/occlusal samples/standard deviation). The proteolytic species belonging to the eight genera are shown in the Y-axis, and the individual sample numbers are shown on the x-axis. On the color scale, tan indicates low relative abundance, and dark brown, a high relative abundance of the given species. The gradient from tan to bright red indicates the z-score of the abundance from low to high. Legends showed the Z-scores, demonstrating the relative abundance levels. Microbial taxon showing a significant difference in abundance between proximal or occlusal niches, are asterisked (*p* ≤ 0.05* and *p* = 0.01**). All species except *A. actinomycetemcomitans* had significantly higher abundance in the proximal rather than the occlusal samples. The latter (marked in red) was the only species with a high abundance in the proximal samples. *p-*value (≤ 0.05) was obtained using *t-*test.

Additionally, we noted a heavy presence of collagenolytic bacteria in our samples, implying the critical role they play in dentine collagen digestion, in the deep caries niches. Thus, in varying proportions, both locales harbored 12 species, belonging to eight genera, namely, *Prevotella, Fusobacterium, Bifidobacterium, Streptococcus, Scardovia, Selenomonas, Veillonella* and *Aggregatibacter* that are known for their collagenolytic attributes. All species except *A. actinomycetemcomitans* was significantly higher in proximal rather than the occlusal samples (Figure 5).

**Figure 6.**
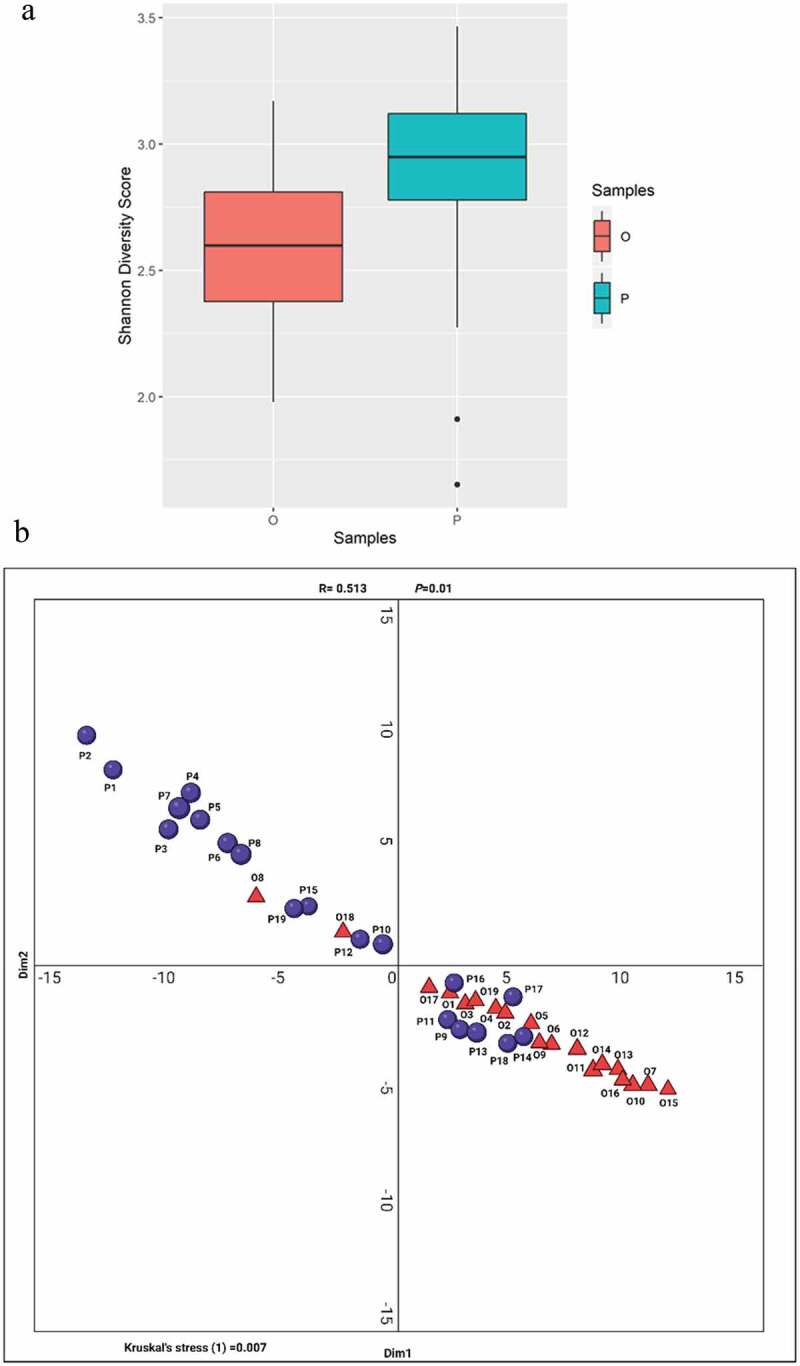
(**a)** Alpha diversity (Shannon Index) of caries-dentin microbial communities. Boxplots of Shannon alpha diversity metrics grouped by sampling site (O: occlusal vs. P: proximal). Box represents the median and interquartile range. Student’s *t*-test identified significant alpha diversity with a *p*-value < 0.05. (b) Bray-Curtis Multidimensional Scaling (MDS) analysis of occlusal and proximal caries microbiota of S-ECC. The plot shows the wider spread of microbiota in proximal caries samples (purple circles) in comparison to the more concentrated clustering of occlusal caries samples in the lower right sector of the plot (red triangles). Analysis of Similarity (ANOSIM) = R = 0.513; *p* = 0.01.

Significant diversity was noted when the overall intra and inter-individual microbial community diversity within the proximal and occlusal samples was measured using Shannon metrics for α and β diversity [37]. The mean species diversity (i.e. α diversity), namely abundance and evenness of the microbiota in the proximal habitats was significantly higher than the occlusal habitats (*p* <0.05), (Figure 6a).

Similar differences were noted in the β diversity of the microbiota between occlusal and proximal cavitated-locales (Figure 6b). Multidimensional Scaling (MDS) of the Bray-Curtis dissimilarity matrix was employed to ascertain differences in the microbial community makeup for each sample. The majority of occlusal samples demonstrated a more analogous microbial community structure, as manifested by their proximity to each other in the MDS plot (Figure 6b). In contrast, in the proximal samples, a more widespread pattern was noted, with a number of outliers reinforcing the diverse nature of the microbiota of the latter niches. In other words, the proximal caries lesions spanned a multivariate space, indicating a compositional microbiome difference within a distinct habitat, as measured by MDS at the species level (stress = 0.007). Furthermore, as can be seen in the MDS plot, the latter difference was significant with a *p-*value of = 0.01 and a high R-value of 0.513 (Figure 6b) [37].

## Discussion

The oral cavity comprises different habitats, such as the teeth, tongue, and gingival sulcus, that provide a complex ecosystem for differential microbial growth [38,39]. The sheltered, cavitated-trenches of dentin-caries lesions, particularly in S-ECC, appear to be a unique habitat in this context [11,40], as reinforced by the complex, polymicrobial consortia noted in the occlusal and proximal cavitated caries milieus in the present study. As far as we are aware, this is the first report to illustrate the richness and the diversity of site-specific microbes of occlusal and proximal caries lesions in S-ECC and compare the compositional differences and diversity of taxa in these two sampled sites.

### Proximal caries lesions harbor a richer and more diverse microbiota than occlusal caries lesions

Using established metrics, Bray Curtis dissimilarity analysis and Shannon diversity indices, the microbiota of occlusal and proximal deep dentinal caries lesions in S-ECC were found to be distinct in terms of the richness of high- or low- abundant taxa and species (Figure 6b). Furthermore, MDS assessment indicated clearly that caries-microbial consortia from the proximal and occlusal samples of the same patient did not typically cluster together.

This phenomenon may be due to the anatomical and structural differences of the occlusal and proximal surfaces of deciduous teeth and/or the intrinsic ecological differences in these two localities. For instance, the occlusal niches are constantly exposed to the ebb and flow of saliva with its arsenal of immune challenges and the masticatory forces due to the intermittent food intake accompanied by incessant tongue movements. Further, it has a much more dynamic environment than the proximal niches in between teeth, which are more sedate and well protected from such extrinsic stresses [8,41]. In addition, the sheltered proximal cavitated locales are almost unreachable to routine oral hygiene measures [8,41], although appliances such as interdental brushes may reach such regions. Thus, it is tempting to speculate that the inherent features overarching the proximal and distal caries ecosystems may be the critical reasons for the significant diversity in the microbiota of these two sites.

### S. mutans and V. alcalescens are the two most prevalent species recovered from all caries sites

We noted that *S. mutans* and *V. alcalescens* were the most prevalent species amongst all evaluated caries sites. There is an ample narrative on the link between *S. mutans* and dental caries due to its superior acidogenic and aciduric potential [13,42,43]. Our data confirms a report by Aas et al. (2008), who also noted that the most prevalent species in either the occlusal or proximal caries of children with S-ECC is *S. mutans* [13]. Another observation that substantiates the work of the latter group [13] is the profusion of *V. alcalescens*, which we noted in both the occlusal and proximal deep-dentine locales (*p* ≥ 0.05). Classically, *Veillonella* spp. and *S. mutans* are known to be co-located and intimately associated with the caries process. The former is thought to nutritionally metabolize the carboxylic acids produced by streptococci, and thereby suppress the cariogenicity of the *mutans*-group of streptococci [44]. On the contrary, others have noted that *V. alcalescens* and *S. mutans* in tandem produce more acids than each of the species separately [45]. *Veillonella* species also easily coaggregate with various oral microbes, including *Streptococcus* spp [46]. thus suggesting a high degree of synergism and mutualism between them [45,47], as was noted here.

### A. parvulum is the most prevalent species in occlusal caries lesions

We noted a highly significant (*p* = 0.01) prevalence of a lactate-producing species, *A. parvulum*, predominantly in the occlusal cavities. Previous studies have also mentioned isolation of the genus *Atopobium* from the carious dentin of children [48,49]. *Atopobium* is not only acidogenic but is also known to be aciduric in nature [49]. The reason why this species predominated in occlusal rather than proximal lesions is unclear but could be due to the aforementioned ecological factors, as described by Kleinberg and Jenkins [50]. They measured the salivary flow and pH in different parts of teeth and observed contrasting pH values even on different surfaces of subjacent proximal teeth, which they surmised would impact the preferential microbial colonization and the eventual propensity for caries [51]. Furthermore, as *A. parvulum* is a predominant inhabitant of the dorsal tongue and saliva, it is plausible that occlusal surfaces with frequent lingual contact and a ready salivary flow may be a preferred colonization site of this organism, in comparison to proximal surfaces.

### An abundance of acidogenic and aciduric microbiota in the proximal cariogenic locale

Detailed analysis of our data indicated the rich and diverse presence (*p* ≤ 0.05) of acidogenic and aciduric taxa, particularly in proximal cavitated niches, in the following order: *P. acidificiens, Leptotrichia* sp., *B. dentium*, and species of genus *Lactobacillus*. This is not surprising as these attributes are essential prerequisites for survival in a very low pH cariogenic niche. Others, too, have reported similar findings. Gross et al. observed *P. acidificiens* in dentinal caries lesions of children in ECC [52], while Obata et al. [48] noted its avidity to dentine collagen, which may be another reason for their preponderance in deep dentinal lesions, in comparison to early enamel caries. Furthermore, Downes and Wade have described the saccharolytic attributes of *P. acidificiens* with the production of acetic, propionic, and succinic acids as end products of dietary carbohydrate metabolism [53]. Others too have confirmed the aciduric potentials of *P. acidificiens* [54,55], a criterion essential for survival in a low pH milieu of deep caries lesions.

Akin to *P. acidificiens*, the genus *Leptotrichia*, a recognized putative cariogen, was noted in our cohorts in significant numbers (*p* ≤ 0.05). Aas et al. have also described the high prevalence of *Leptrotrichia* in deep-dentine caries of deciduous teeth [13] which are known to ferment many mono- and disaccharides to lactic acid [56].

The acidogenic and aciduric *Bifidobacteriaceae*, which play a contributory role in caries progression [24], was also highly prevalent in proximal cavities (*p* < 0.05). In line with our observation, Becker et al. identified *Bifidobacterium* species as the most prevalent cariogen that even outnumbered *S. mutans* in dentinal caries of children [57]. Furthermore, in a very early *in vitro* study, Van Houte et al. have shown that bifidobacteria had the potential to reduce the pH of glucose-supplemented media, upto to < 4.2 [58], adequate for demineralization of both enamel and dentine [59].

Historically, the two major cariogens were considered to be *mutans*-group streptococci and lactobacilli, and the latter is particularly known to be found in the advancing front of the dentinal caries lesions [44]. Indeed, lactobacilli are a critical secondary pathogen in dental caries [21,60]. Hence it is not surprising that we noted a spectrum of 22 *Lactobacillus* species in the dentine caries samples of both the proximal and occlusal lesions. Of these, three species, *Lactobacillus salivarius, L. gastricus*, and *L. ghanensis* stood out as significantly more prevalent in the proximal cavities (*p* ≤ *P. denticolens*

0.05) (Figure 2a).

Apart from the foregoing predominant genera, less well-known others were noted in significant proportions in the proximal cavitated niches (*p* = 0.01). These were *Actinobacteria* belonging to *Bifidobacteriaceae*, namely *P. denticolens* and *S. inopinata*. Supportive of these findings, Mantzourani et al. have also identified *B. dentium, P. denticolens*, and *S. inopinata* from the caries samples of primary teeth [61]. An intriguing characteristic shared by these species is their ability to degrade complex carbohydrates, including dextran [62] which potentiates the production of demineralizing acids within the cariogenic biofilm, even in the absence of fermentable carbohydrates [62]. Furthermore, another recent study has reported the synergism in acidogenicity in dual-species biofilms of *P. denticolens, S. inopinata*, and *B. dentium*, with *S. mutans* [63]. Taken together, it is clear that such microbial consortia play a decisive role in the pathobiology of deep caries lesions, in particular.

### Several other attributes of the isolates mediate cariogenicity in S-ECC

There are several major pathogenic attributes of the putative cariogenic microbiota that make them fit for their role and thus stand out as key cariogens, especially in deep dentinal lesions. These include, apart from their acidogenic and aciduric potential, potency to adhere and colonize both enamel and dentinal surfaces, collagenolytic and proteolytic potential to degrade dentinal collagen, and ureolytic properties that assist degradation of metabolic urea of the biofilm microbiota. Some or most of these attributes are noted in the predominant species in both occlusal and proximal caries in this study.

Dentin tissue is essentially a hydroxyapatite mineral crystallite collagen matrix [64]. Indeed, type I collagen comprises up to 90% of the organic material of the extracellular dentinal matrix [65]. Cariogens such as *S. mutans* and *B. dentium* can preferentially colonize dentin as they possess adhesins (SpaA adhesins) that mediate their attachment to collagen [66,67], while the former has additional collagen-binding proteins Cnm and Cbm [68,69]. Hence, the duality of key attributes, their adhesive and acidogenic nature, makes *S. mutans* eminently suitable to be a key cariogen, particularly in dentinal caries.

As opposed to the richness of acidogenic cariogens such as *S. mutans*, we also noted a significant proportion of health-associated ureolytic species *C. matruchotii, Haemophilus parainfluenzae*, and *A. naeslundii* in varying proportions in both the proximal and occlusal samples. *C. matruchotii* had been suggested as a nucleating species of the biofilm bacterial community in several studies. Welch et al. [70] noted a multi-genus conglomerate of nine taxa structured around filamentous corynebacterial cells. *C. matruchotii*, in particular, raises the biofilm pH by using acetate and lactate produced by the acidogenic plaque microbes [70]. Furthermore, significant numbers of other ureolytic species such as *H. parainfluenzae* and *A. naeslundii* were also noted in our samples, as was earlier reported by Ma et al. in S-ECC plaque [71]. The profusion of these urease-producing taxa amongst the abundant acidogenic, aciduric microbial consortia, particularly in the proximal deep-dentine milieu, is intriguing. It may be an attempt at keeping the pH to moderate survivable levels of the biofilm community. However, further studies are required to explore their role in such consortia.

It appears that microbe-mediated acidification of the plaque biofilm is not just the prime mover of caries progression [72]. The acidic milieu, in turn, can activate endogenous dentin-embedded and salivary matrix metalloproteinases (MMPs) and cysteine cathepsins, which are thought to play a significant role in caries development [64,73] as proposed by Takahashi and Nyvad [72].

For instance, the trio, *Prevotella nigrescens, Fusobacterium nucleatum*, and *A. actinomycetemcomitans* produce both intra- and extracellular gelatinolytic proteinases that may activate latent pro-MMP-9 [74]. Furthermore, in our study, *F. nucleatum* was detected in both proximal and occlusal lesions. The latter is a core constituent of dental biofilms and plays a pivotal role in bridging microbes of early and late colonizing species [75]. Thus, despite their relatively low numbers, the synergistic impact of the proteases of the trio, *F. nucleatum, A. actinomycetemcomitans*, and *P. nigrescens*, may significantly contribute to the structural disintegration of the collagenous scaffold of the dentine, in tandem with other collagenase producing species of *Prevotella*.

Several studies have reported the high prevalence of several *Prevotella* spp., particularly in dentine caries [22,49,72,76]. Indeed, this led Teng et al. to suggest the relative abundance of a panel of seven keystone *Prevotella* spp. from caries lesions of ECC [25,77]. Our findings, with a total of 27 species belonging to the genus *Prevotella*, with 23 species present at both the occlusal and proximal sites, and four species (*Prevotella intermedia, Prevotella nanceiensis, Prevotella marshii*, and *Prevotella fusca*) only at the proximal sites, confirm the assertions of Teng et al. [25]. Others too have echoed these sentiments and surmised that the overexpression of collagenases by *Prevotella* species during proteolytic metabolism might significantly contribute to the progression of dental caries especially at the advancing dentinal front [76]. The rich aggregates of the *Prevotella* species with such collagenolytic attributes that were recovered from our samples included *P. denticola, P. histicola, P. melaninogenica, P. multisaccharivorax*, and low prevalent, *P. nigrescens*, and *P. intermedia*. Indeed, all of these species have been previously recorded by others as isolates from cariogenic lesions [78–82] (Figure 5). Our findings, therefore, further substantiate the view that *Prevotella* species play a leading role in proteolytic digestion and the progression of dentinal caries. However, further work is needed to ascertain the specific mechanisms by which they mediate such changes.

### Collagenolytic microbes are highly prevalent in S-ECC (Figure 5)

Although the acidogenic and aciduric attributes of *S. mutans* are well known, their ability to degrade human collagen (acid-soluble, type I) – the major constituent of dentine, by their extracellular proteases is poorly recognized. Some studies including, new metabolomic research, indicate the overexpression of collagenase gene expression in *S. mutans* in dental caries [79,83]. As mentioned above, we had a rich harvest of *S. mutans* across all caries samples, albeit with a significant preponderance in the proximal niches. Their heavy presence in deep dentinal caries where collagen is plentiful appears to be a likely reflection of the tenacity and avidity of *S. mutans* for collagen and fibronectin, as well as the abundance of peptidases and collagenases they possess [76]. Finally, in this context, a range of several other cohabitant microbes with known collagenolytic attributes [67,79,80,83,84] were also isolated from dentin eco-niches. They were, *B. dentium, S. inopinata* of the phylum *Actinobacteria, S. noxia* and *V. parvulum* [79]. These consortia involved in initiation and degradation of the demineralized organic matrix of the dentinal tissues, and the activation of host-derived proteases ratifies and add credence to the ecological hypothesis of dentine proposed by Takahashi and Nyvad [72], as well as the tissue-dependent caries propagation hypothesis of Simon-Soro et al. [76] which states that while acid-producing bacteria are the prime movers of enamel penetration, the dentin degrading collagenolytic organisms which destroy deeper dentinal tissues are co-contributors in caries propagation.

Hence, if not intervened and appropriately treated, S-ECC may perhaps have far-reaching effects, even extending to adulthood, and impact the general health of these children. Inquimbert et al. [85] and a few others [86–88] have shown that early colonization of ECC lesions by periodontopathic species may be construed as a marker of periodontal disease risk later in life. In the investigation, they identified a number of periodontopathic organisms such as *C. gracilis, S. noxia*, and *P. intermedia* known to be associated with the initiation and progression of periodontal infection from ECC lesions [85–88].

We also noted several pathogens such as *C. concisus, C. granulosa, Neisseria bacilliformis*, and *G. adiacens*, implicated in the oral-systemic disease axis, amongst the caries microbiota. The two former organisms (*C. granulosa, N. bacilliformis*) are implicated in abscess development and bacteremia secondary to focal infections [89,90]. In addition, the oral *C. concisus* strains have been associated with human irritable bowel syndrome [91,92]. Furthermore, members of the ‘HACEK’ group bacteria, i.e. *Haemophilus* sp., *Aggregatibacter* sp., *C. hominis, E. corrodens, Kingella* sp., and *G. adiacens*, a nutritionally variant streptococcus, all known to cause bacterial endocarditis [93,94], were also prevalent in the dentinal caries samples. Thus, it is tempting to speculate that reservoirs of these microbes within cavitated lesions of S-ECC may act as potential reservoirs that may contribute to the foregoing systemic diseases in these children.

### Limitations of the study

Our study has some limitations. The current data were derived from a relatively small sample of children and need to be confirmed in a larger cohort, ideally from another geographic locale. Further, our report encompasses species composition of deep caries lesions in general but does not appertain or relate to a specific depth of the lesion. Therefore, future studies are required to evaluate the microbiome composition in dentinal cavities of varying depths to clearly understand the natural history of S-ECC and the compositional variations of the microbiota during lesion progression towards the pulpal axis.

### Conclusions and Future Research Directions

As noted, distinct differences exist between the caries microbiota of occlusal and proximal caries in S-ECC. An array of collagenolytic organisms were found in both occlusal and proximal lesions indicating their critical role in the pathobiology of deep dentinal caries in S-ECC. The clinical implications of these findings in terms of the rate and severity of caries progression, and the interventional approaches remain to be determined. In addition, it will be instructive to compare the site-specific caries microbiota of healthy enamel plaque to differentiate the possible bacterial eco-communities in health and disease. In addition, the detailed microbial composition of lesion-free, proximal and occlusal surfaces of teeth are needed to determine the impact of salivary flow rate on the microbiome of these niches.

## Data Availability

Data of the present study were evaluated and presented in this article. Additional datasets, if required, can be provided by the corresponding author on reasonable request.
